# Prefrontal cortical morphometric covariance and cognitive task performance in healthy young adults: an exploratory analysis of Human Connectome Project data

**DOI:** 10.3389/fnhum.2026.1976371

**Published:** 2026-10-22

**Authors:** Roman Chivers

**Affiliations:** Seattle Preparatory School, Seattle, WA, United States

**Keywords:** cognitive control, human connectome project, inhibitory control, orbitofrontal cortex, prefrontal cortex, structural covariance, working memory

## Abstract

The prefrontal cortex (PFC) is implicated in many cognitive tasks, including working memory, reward-based decision-making, executive control, and metacognition. This exploratory analysis investigated whether individual expression of PFC cortical thickness and surface area covariance components is associated with cognitive task performance. The sample comprised Human Connectome Project (HCP) MRI data from 743 healthy adults (age 22–36+). Oblimin-rotated principal component analysis (PCA) was applied to residualized thickness and surface area for 22 PFC regions, controlling for age, sex, and ICV. Component scores were entered as predictors in separate regression models for the Flanker Age-Adjusted Score (inhibitory control), the Gambling RT Difference (RT for the same “larger” guess in reward minus punishment blocks), and 2-Back median RT (working memory speed). Parallel analysis favored two thickness components; a prespecified three-component solution (Global/Lateral PFC, RC1; Orbitofrontal, RC2; Anterior Cingulate, RC3; 53.7% of variance) was retained by decision, and RC1 and RC2 were only moderately stable across bootstrap resamples. RC1 was nominally associated with lower Flanker scores (β = −0.103, *p* = 0.027), opposite to the predicted direction, and RC2 with a smaller Gambling RT Difference (β = −0.103, *p* = 0.025); neither survived false discovery rate (FDR) correction (*q* = 0.054). In uncorrected models added at review, whole-cortex mean thickness alone robustly predicted Flanker scores but not the Gambling RT Difference; neither component association survived regressing mean thickness out before PCA. In surface area analyses, greater orbitofrontal surface area (AC2) was associated with faster 2-Back RT (β = −0.125, *p* = 0.003; *q* = 0.032 across 12 surface area tests and 0.064 across all 24 component tests), not explained by a speed-accuracy tradeoff. This unpredicted association persisted in most sensitivity analyses but was attenuated and non-significant when total surface area was regressed out before PCA (β = −0.065, *p* = 0.078). In conclusion, no prefrontal-specific association was robust. Global cortical thickness accounts for the Flanker association more parsimoniously, the Gambling RT Difference association was not robust under either account, and the orbitofrontal surface area association could not be separated from global surface area; all associations require replication.

## Introduction

The prefrontal cortex (PFC) occupies a central place in cognitive neuroscience. The PFC has evolved more than any other cortical region in the primate lineage, and its functions underpin the core regulatory processes that characterize adaptive human behavior: response inhibition, reward-versus-cost evaluation, error detection, and working memory. Evidence from lesion, functional neuroimaging, and developmental studies is broadly consistent with this view. However, the exact structural parameters of the PFC, i.e., its morphology, cortical thickness, surface area, as well as the relationships among them, that relate to the performance of its associated cognitive functions in healthy adults, remain to be elucidated. The PFC is not one thing; the PFC is a confederation of functionally and cytoarchitecturally heterogeneous regions with distinct contributions to cognition. In terms of overall function, the lateral PFC may be broadly divided into the superior, middle, and inferior frontal gyri. Working memory and response inhibition are general themes of the lateral PFC, with the dorsolateral prefrontal cortex being heavily implicated in tasks requiring attention-gated manipulation of information held in mind (Aron et al., 2014; Curtis and D'Esposito, 2003). The orbitofrontal cortex (OFC) and its ventromedial extension are involved in encoding expectations of future outcome (reward value) and in the integration of past feedback to guide the calibration of new choices following a shift in reward schedules of reinforcement (O’Doherty et al., 2001; Wallis, 2007). Finally, the anterior cingulate cortex (ACC) on the medial surface of the cortex tends to be activated wherever there is some indication of a problem or strain, such as when there is conflict or the person needs to register that their current strategy is not succeeding (Botvinick et al., 2001; Fleming and Dolan, 2012). The PFC may therefore be understood as a confederation of functionally distinct sub-networks, each engaged in somewhat different activities yet sharing a common underlying structural organization. These regions differ in their functional specializations and developmental trajectories.

The function of individual PFC subregions has been extensively investigated, but the relation of their structural organization (i.e., the pattern of covariation of their morphological characteristics) to their function is unclear. Structural covariance is thought to have several biological sources, alongside measurement factors such as shared reconstruction error. Alexander-Bloch et al. (2013) suggested that part of the structural covariance between brain regions arises from the coordination of their developmental timing, with regions whose developmental timing is coordinated tending to retain coordinate morphological organization in adulthood. Mechelli et al. (2005) suggested that part of the structural covariance between brain regions in adults arises from the use-related activity between the regions, with regions having coordinate activity tending to have coordinate morphological organization. Both explanations are plausible in this sample of young adults. Structural covariance is also a population-level property: it describes how morphology covaries across individuals and does not directly measure structural connectivity within any one person.

Genetic and environmental factors that influence brain development are not evenly distributed across the different dimensions of brain structure. In particular, cortical thickness and surface area have largely independent genetic influences (Panizzon et al., 2009). Thickness is more related to post-migratory processes such as post-mitotic neuronal differentiation, including myelination, dendritic trees, and synapses (Rakic, 1988; Wierenga et al., 2014). Surface area is more related to prenatal processes and is primarily determined by the number of radial units that are produced by the cortical progenitors through proliferation (Raznahan et al., 2011). Therefore, thickness and surface area structural covariance networks are expected to carry different and dissociable information about brain function. However, so far, thickness and surface area are often analyzed separately, and the morphological organization of the whole PFC and how different sub-regions covary is rarely investigated.

Previous studies investigating the relationship between PFC structure and cognition have yielded largely inconclusive results. While previous studies have shown that alterations in the structure of the PFC (such as cortical thinning reflecting processes of synaptic pruning and myelination) are associated with intelligence, few studies have investigated the PFC in depth. Shaw et al. (2006) showed in a longitudinal study of children and adolescents that the rate of cortical thinning in the PFC was a better predictor of intelligence than any given measurement of cortical thickness at a specific time point. In adults, in whom the process of active pruning is largely completed, the relationship is less clear. Several studies have linked relatively thinner PFC cortices to better performance on tasks of executive function (Tamnes et al., 2010), although the biological basis of this association in adulthood is uncertain. With respect to OFC structure, Schoenbaum et al. (2009) have identified the OFC as a region crucial to the evaluation of monetary and non-monetary value and to learning to switch behaviors in response to changing contingencies of reward. Fleming and Dolan (2012) reviewed neuroimaging and lesion evidence linking anterior PFC structure to metacognitive monitoring accuracy (i.e., accuracy in assessing one’s performance). These studies are important, but as with all the previous studies focusing on individual regions and cognitive measures, none have investigated the structural covariance (i.e., the population-level pattern of covarying morphology) of the PFC and its relationship to cognitive performance in multiple cognitive domains. It is the last question that is explored in the present study.

This exploratory analysis examined whether individual expression of population-level structural covariance components within the PFC is associated with variation in cognitive performance. Structural MRI data from participants in the Human Connectome Project (HCP) data set were analyzed (Van Essen et al., 2013). PFC was analyzed by applying a principal component analysis (PCA) to the 22 residualized cortical thickness and 22 residualized surface area variables. The component scores were used to predict performance on three primary cognitive measures: Flanker task, which is a measure of inhibitory control; the Gambling task, which yields a measure of response timing across reward and punishment blocks; and the 2-Back task, which is a measure of working memory speed. A block-level choice score derived from the gambling task, intended to test H4, is reported in the Supplementary material. Seven hundred forty-three healthy adults were included in the HCP data set after cleaning.

The study aims to achieve five main goals: (1) Investigate structural covariance networks in the PFC through thickness and surface area measurements. (2) Assess if thickness networks in the lateral PFC predict inhibitory control. (3) Analyze the influence of OFC structure on response timing across reward and punishment blocks. (4) Explore whether joint expression of lateral PFC and ACC components predicts feedback-contingent choice behavior, and (5) Determine if PFC structure predicts the speed of working memory functions. Five hypotheses were proposed: (H1) PFC cortical thickness will fractionate into three structural covariance networks related to lateral executive, orbitofrontal-evaluative, and cingulate monitoring systems; (H2) improved inhibitory control on the Flanker task is associated with greater expression of the lateral PFC thickness component; (H3) quicker working memory response times are also linked to this lateral PFC component; (H4) increased win-stay/lose-switch sensitivity is associated with the interaction between lateral PFC and ACC component expression; and (H5) greater expression of the OFC thickness component predicts more balanced reward-punishment decision behavior. H4 and H5 were formulated under readings of the gambling task variables that proved incorrect (see Cognitive Outcome Variables), so neither could be tested as stated.

## Materials and methods

The data are the HCP Young Adult open-access tabular data (1,206 participants; the S1200 sample), exported from ConnectomeDB powered by BALSA (HCP-Young Adult 2025 release) on 5 October 2026. The 2025 release updated fMRI processing; non-imaging data are unchanged (Human Connectome Project, 2025). This dataset maps brain connectivity and behavior in healthy adults. The study was approved by the Washington University IRB, and all participants gave written consent.

### Participants

Originally, there were 1,113 healthy young adults aged 22 to 36+. Participants were included if the Full 3T Imaging Protocol Completed field (3T_Full_MR_Compl) was true and the Coded QC Issue field (QC_Issue) was empty. After that filtration, 764 participants with complete demographic and structural data were retained. In the next step, cases with completely missing values on any analysis variable were removed. Missing data in gambling reaction time variables arose because participants who never guessed “larger” within a block type have no defined median RT for that cell. This reflects their task behavior, not random dropout. Thus, the final sample was 743 adults, representing a 97.3% retention rate of the eligible sample (see Table 1). The HCP sample includes monozygotic and dizygotic twins and non-twin siblings, so participants are not fully independent. This analysis used the HCP open-access data release, which does not include family identifiers, years of education, or exact age; these variables are available only in the HCP Restricted Data release.

**Table 1:** Missingness summary by variable block.

Variable block	*N* missing	% Missing	Notes
FreeSurfer Thickness (22 vars)	0	0.00%	Complete for all 764 participants
FreeSurfer Surface Area (22 vars)	0	0.00%	Complete for all 764 participants
Flanker Age-Adjusted Score	0	0.00%	NIH Toolbox, none missing
WM 2-Back Median RT	5	0.65%	Task non-completion
Gambling RT: Reward Larger	17	2.23%	Never guessed “larger” in reward blocks
Gambling RT: Punish Larger	11	1.44%	Never guessed “larger” in punishment blocks
Gambling Perc: Reward Larger	0	0.00%	None missing
Gambling Perc: Punish Smaller	0	0.00%	None missing
Age / Sex / ICV	0	0.00%	Complete for all participants
TOTAL excluded (any missing)	21	2.75%	Final N = 743 retained

WM, working memory; RT, reaction time; Perc, percentage of choices; ICV, intracranial volume; NIH, National Institutes of Health. No responses were missed in either gambling block type. 2-Back accuracy, used in sensitivity analyses, was available for all 743 participants.

### Neuroimaging data acquisition

Structural MRI data were acquired at Washington University on a customized 3 T Siemens Skyra scanner. The cortical and subcortical data were extracted using the 3D T1-weighted MPRAGE sequence: TR = 2,400 ms, TE = 2.14 ms, TI = 1,000 ms, flip angle = 8°, voxel size = 0.7 mm isotropic, Field of View (FOV) = 224 × 224 mm. The cortical reconstruction and volumetric segmentation were done with FreeSurfer 5.3.0-HCP in the HCP minimal preprocessing pipelines (Glasser et al., 2013); the regional thickness, surface area, and ICV values used here are the FreeSurfer summary statistics included in the open-access tabular data, which were identical in exports made on 2 and 5 October 2026. The data were parcellated to the Desikan-Killiany atlas to obtain thickness and surface area of 11 prefrontal regions per hemisphere (22 variables in total): superior frontal, caudal middle frontal, rostral middle frontal, pars opercularis, pars orbitalis, pars triangularis, lateral orbitofrontal, medial orbitofrontal, frontal pole, rostral anterior cingulate, and caudal anterior cingulate.

### Structural variables and covariate residualization

Prior to PCA, 22 bilateral prefrontal cortical thickness variables and 22 surface area variables were residualized to remove age, sex, and ICV as nuisance variables. To do this, the z-scored values of each of the structural variables were regressed on the scalar value of age corresponding to each of the four HCP age groups (23.5, 28, 33, 38), sex, where 0 = female and 1 = male and intracranial volume (FreeSurfer ICV). Residualization was performed in the final analysis sample (*N* = 743). Because residualizing thickness on ICV is not standard, results with ICV omitted are reported in Supplementary Tables S3, S4. The residuals of each of these regressions were retained as the residualized structural variables that were used in the PCA. Subsequently, the residualized structural variables were entered as predictors in each of the models of cognitive performance along with age and sex as additional covariates; ICV was not included in these analyses as it was already partialed out in the residuals of the structural variables used as predictors.

The two adjustments for age and sex serve different purposes. Residualization removes age-, sex-, and ICV-related variance from the structural predictors, so that component scores are approximately uncorrelated with these variables, but it does not remove age- or sex-related variance from the cognitive outcomes. Including age and sex as covariates in the outcome models absorbs that variance, which reduces residual error and improves the precision of the structural estimates, and allows the independent effects of age and sex on each outcome to be reported. Because the Flanker score is already age-adjusted, the age term in the Flanker model is largely redundant; it was retained so that all models share the same covariate set.

### Principal component analysis

To gain insight into the underlying structural networks within PFC, independent principal components analyses (PCAs) were conducted on the 22 residualized thickness variables and 22 residualized surface area variables using the psych package in R (Revelle, 2024). Parallel analysis (Horn, 1965) indicated two components (the third observed eigenvalue, 1.21, fell just below the mean random eigenvalue, 1.23) and the scree criterion three; three were retained by decision because that solution had been specified *a priori* (H1). Oblimin rotation was used as the rotation method to allow for correlation between components as prefrontal sub-regions are anatomically contiguous and are therefore expected to share vascular pathways. The component scores derived from this analysis were used as structural predictors in subsequent regression analyses. Each participant’s component score reflects the degree to which that individual expresses a covariance pattern defined across the whole sample; it does not measure structural connectivity specific to that individual. The inter-component correlations derived from the oblique solution are also reported to further characterize the relationships between components within the PFC. Stability of the thickness solution was assessed in 1,000 bootstrap resamples using Tucker’s congruence coefficient (Lorenzo-Seva and ten Berge, 2006); in each resample, bootstrap components were matched one-to-one to the full-sample components by the permutation that maximized total absolute congruence. Factor loading heatmaps for both the thickness and surface area solutions are provided in Figure 1.

**Figure 1:**
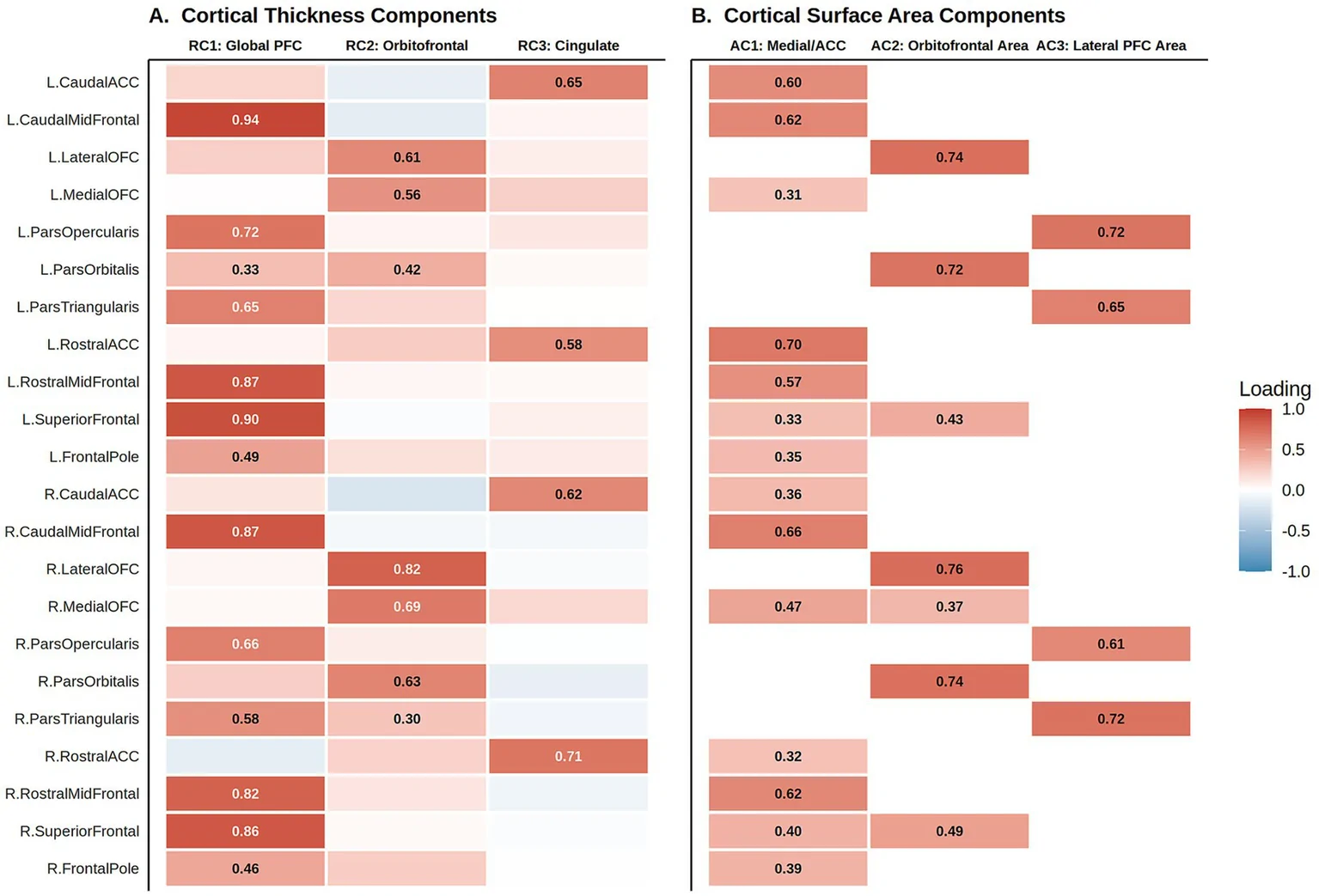
PCA factor loading heatmaps. **(A)** Cortical thickness components (RC1–RC3). **(B)** Surface area components (AC1–AC3). Values shown where |*λ*| ≥ 0.30. Loading magnitude is reflected by color intensity; bold loadings indicate primary component assignment. PCA, Principal component analysis; RC, Rotated cortical thickness component (RC1, Global/Lateral PFC; RC2, Orbitofrontal; RC3, Anterior Cingulate); AC: Rotated surface area component (AC1, Medial/Cingulate; AC2, Orbitofrontal; AC3, Lateral Inferior Frontal); PFC, Prefrontal cortex; *λ*: Component loading.

### Cognitive outcome variables

To elucidate the pattern of relations underlying these measures, three primary outcomes were analyzed, with the Block Choice Score and 2-Back accuracy examined in secondary analyses. The variables were: (1) Flanker Age-Adjusted Score: the age-adjusted scores derived from the NIH Toolbox Flanker Inhibitory Control and Attention Test (Zelazo et al., 2013). These scores are age-adjusted to represent relative inhibitory function, given the typical performance expected of individuals of a given age. They are not adjusted for education, which is not available in the open-access release. (2) Gambling RT Difference: median RT on trials in which the participant guessed “larger” in reward blocks minus the same measure in punishment blocks, on the HCP gambling task adapted from Delgado et al. (2000) and Barch et al. (2013). “Larger” refers to the participant’s guess, not the size of the outcome (Human Connectome Project, 2017). Because block type is not cued before each guess, the score compares response speed for the same choice across block contexts and is interpreted as indexing the carryover of recent outcome context into response speed. The “larger” cells were selected before analysis; because no principled reason favors them over the “smaller” cells, results for “smaller” trials are reported in Supplementary Table S1. As a difference between two correlated reaction time measures, this score is expected to have lower reliability than either component measure (Hedge et al., 2018). (3) Block Choice Score (BCS), intended to test H4: the proportion of “larger” guesses in reward blocks plus the proportion of “smaller” guesses in punishment blocks (Gambling_Task_Reward_Perc_Larger and Gambling_Task_Punish_Perc_Smaller; range 0–2; no responses were missed in either block type in the analysis sample). Because block type is not cued and outcomes are predetermined by block (Barch et al., 2013), the BCS cannot index trial-level win-stay/lose-switch behavior, so H4 could not be tested; the BCS models are reported in Supplementary Tables S5, S6. (4) 2-Back Median Reaction Time: the median RT on correct trials for the 2-back working memory n-back task, adapted from Barch et al. (2013). This measures the RT associated with processing n-back information within working memory. 2-Back accuracy (WM_Task_2bk_Acc) was examined in secondary analyses added at review, as a covariate and as a parallel outcome (Supplementary Tables S4, S7).

### Statistical analysis

These models were estimated using multiple linear regression for each cognitive outcome, regressing the PFC structural component scores (RC1, RC2, RC3 for thickness measures; AC1, AC2, AC3 for area measures) onto the respective cognitive outcome, including age and sex as covariates. The standardized beta coefficients (β) were derived using the lm.beta package. The partial eta-squared (η^2^p) effect sizes were computed from the t statistics as t^2^/(t^2^ + df), equivalent to Type III partial η^2^ for single-degree-of-freedom terms. The joint expression of the lateral PFC and ACC components that was predicted to explain win-stay/lose-switch sensitivity (H4) was investigated by entering the product interaction term (RC1 × RC3) into an augmented model. All models were assessed for significance at α = 0.05. Normality of residuals was assessed with Shapiro–Wilk tests and Q-Q plots (Supplementary Figures S1, S2). Homoscedasticity was assessed with the Breusch-Pagan test from the lmtest package (Zeileis and Hothorn, 2002) and Residuals vs. Fitted plots (Supplementary Figures S2, S3). Assumption diagnostics for eight regression models (the three primary outcomes plus the Block Choice Score, each for thickness and for surface area) are summarized in Table 2. Multicollinearity among predictor variables was assessed with Variance Inflation Factors (VIF). VIF values for the cortical thickness models are presented in Table 3. All figures were generated with ggplot2 (Wickham, 2016). To control for multiple testing, Benjamini–Hochberg false discovery rate (FDR) correction (Benjamini and Hochberg, 1995) was applied to the component-level *p* values. The primary family comprised the four directional hypothesis tests: H2 (RC1 → Flanker), H3 (RC1 → 2-Back RT), H4 (RC1 × RC3 → BCS), and H5 (RC2 → Gambling RT Difference). This family was retained as prespecified for transparency, although H4 and H5 could not be tested as worded (see Cognitive Outcome Variables); this is the conservative choice, because excluding the H4 test would reduce q for H2 and H5 from 0.054 to 0.040. As broader checks, correction was applied across the 12 thickness component tests, the 12 surface area component tests (three components × four outcomes each), and all 24 component tests; the surface area family, and therefore the 24-test family, includes the tests for the Block Choice Score, which is reported in the Supplement. For 2-Back accuracy, *q* values were computed across its six component tests (three thickness and three surface area). FDR-adjusted values are reported as q, and the analyses are treated as exploratory. For the Flanker and Gambling models, sensitivity analyses added mean cortical thickness as a covariate, computed as the area-weighted mean across all 68 Desikan-Killiany regions (the sum of regional thickness × regional surface area, divided by total surface area); regressed mean thickness out of the regional thickness measures before repeating the PCA; re-estimated the models with HC3 standard errors (sandwich package; Zeileis, 2004), with robust M-regression using Tukey’s biweight (MASS package; Venables and Ripley, 2002), and after excluding cases with Cook’s distance > 4/N; and used the unadjusted Flanker score with age as a covariate (Supplementary Table S3). The same analyses were applied to the AC2 association with 2-Back RT, with total cortical surface area (the sum of the 68 regional surface areas) in place of mean thickness, including regressing total surface area out of the regional surface area measures before repeating the PCA, and 2-Back accuracy was added as a covariate and examined as a parallel outcome (Supplementary Tables S4, S7). Mean thickness and total surface area were entered without residualization, alongside age and sex; results were similar when mean thickness was first residualized on age, sex, and ICV. Each global measure was also entered without the components, alongside age and sex, and mean thickness alone was re-estimated with robust regression. These sensitivity and secondary analyses were added at review and were not prespecified. Analyses used R 4.3.3 (R Core Team, 2024) with psych 2.4.1 (Revelle, 2024), GPArotation 2024.2–1 (Bernaards and Jennrich, 2005), lm.beta 1.7–3 (Behrendt, 2025), car 3.1–2 (Fox and Weisberg, 2019), lmtest 0.9–40 (Zeileis and Hothorn, 2002), sandwich 3.1–0 (Zeileis, 2004), and MASS 7.3–60 (Venables and Ripley, 2002).

**Table 2:** Assumption check summary across the eight regression models.

Model	Breusch-Pagan *p*	Shapiro–Wilk *p*
Flanker: RC1 + RC2 + RC3 + Age+Sex	0.568	<0.001
Gambling RT: RC1 + RC2 + RC3 + Age+Sex	0.564	<0.001
BCS: RC1 + RC2 + RC3 + Age+Sex	0.070	<0.001
2-Back RT: RC1 + RC2 + RC3 + Age+Sex	0.484	0.034
Flanker: AC1 + AC2 + AC3 + Age+Sex	0.611	<0.001
Gambling RT: AC1 + AC2 + AC3 + Age+Sex	0.323	<0.001
BCS: AC1 + AC2 + AC3 + Age+Sex	0.141	<0.001
2-Back RT: AC1 + AC2 + AC3 + Age+Sex	0.715	0.033

RC1–RC3, rotated cortical thickness components (RC1, Global/Lateral PFC; RC2, Orbitofrontal; RC3, Anterior Cingulate); AC1–AC3, rotated surface area components (AC1, Medial/Cingulate; AC2, Orbitofrontal; AC3, Lateral Inferior Frontal); PFC, prefrontal cortex; RT, reaction time; BCS, Block Choice Score.

**Table 3:** Variance inflation factors (VIF) for the cortical thickness regression models.

Predictor	Flanker model	Gambling RT	2-Back RT model
RC1	1.64	1.64	1.64
RC2	1.58	1.58	1.58
RC3	1.10	1.10	1.10
Age (numeric)	1.03	1.03	1.03
Sex (binary)	1.03	1.03	1.03
Maximum VIF	1.64	1.64	1.64

VIF, variance inflation factor; RC, rotated cortical thickness component; RT, reaction time; BCS, Block Choice Score. Component definitions as in Table 2. Component scores correlated |r| < 0.001 with age and sex. With mean cortical thickness added to the Flanker and Gambling models, VIFs were 4.00 (RC1), 1.81 (RC2), 1.15 (RC3), 1.12 (age), 1.05 (sex), and 4.90 (mean thickness); with mean thickness entered without the components, all VIFs were ≤ 1.05.

Because family identifiers were not available (see Participants), family structure could not be modeled with linear mixed-effects models, generalized estimating equations, or exchangeability-block permutation tests (Winkler et al., 2015). Treating related participants as independent violates the ordinary least squares assumption of independent errors. Because both the predictors and the outcomes are heritable, ignoring family clustering likely makes the reported p and *q* values anti-conservative, although this is not guaranteed for every coefficient.

## Results

### Descriptive statistics and missingness

Missingness rates for the complete-case sample (*N* = 743) are presented in Table 1. Missingness was highest for the gambling RT variables: Gambling RT Reward Larger (2.23%) and Gambling RT Punish Larger (1.44%), arising because participants who never guessed “larger” within a block type have no defined median RT for that cell. Flanker scores had no missing values, and 2-Back RT was missing for 0.65% of participants. Given that FreeSurfer structural variables were available for all 764 participants in the screened sample, missingness rates for these variables were zero. The 21 participants with missing data (2.75% of the screened sample) were removed by listwise deletion.

### Statistical assumption checks

All models were subjected to a standard set of regression diagnostics. In this regard, Breusch-Pagan (homoscedasticity) and Shapiro–Wilk (normality) test results are given for each of the eight models (the three primary outcomes plus the Block Choice Score, each for thickness and surface area) in Table 2. Neither of the two primary significant models exhibited violations of homoscedasticity as indicated by the Breusch-Pagan test (Flanker: *p* = 0.568; Gambling RT: *p* = 0.564). Shapiro–Wilk tests indicated non-normal residuals in all models (all *p* < 0.05), consistent with the ceiling tendency of the Flanker score and the heavy tails of the RT difference score. With *N* = 743, ordinary least squares estimates are robust to moderate non-normality, and the sensitivity analyses with HC3 standard errors and robust regression address this directly (Supplementary Table S3). Q-Q plots are presented in Supplementary Figure S1; regression diagnostic plots for the Flanker model are displayed in Supplementary Figure S2, and for the Gambling RT model in Supplementary Figure S3. None of the eight models showed significant heteroscedasticity (all Breusch-Pagan *ps* ≥ 0.07).

Multicollinearity diagnostics are presented in Table 3. As observed, none of the VIF values exceeded 1.7 in any of the models, well below the conventional thresholds of 5 and 10. The VIF values are the same for all models because the set of predictor variables that are entered into each equation consists of the same variables (RC1, RC2, RC3, age and sex). Although the inter-component correlations that resulted from the oblimin rotation (*r* = 0.22–0.60) indicated some modest degree of interrelation between the components, they were not sufficient to give rise to a problematic level of multicollinearity. The component structure is therefore satisfactory for regression analysis. With mean cortical thickness added to the Flanker and Gambling models, the VIFs rose to 4.00 for RC1 and 4.90 for mean thickness (Table 3 note).

### Principal component analysis: structural covariance networks

#### Cortical thickness components

PCA of the 22 residualized prefrontal thickness variables, as applied to the study sample, revealed three components that collectively explained 53.7% of the variance (Table 4). These components and their constituent variables are described in detail with SS loadings, % variance, cumulative % variance and with the primary region contributing to each variable. The factor loading heatmap for this analysis is depicted in Figure 1A.

**Table 4:** Cortical thickness PCA: component summary.

Component	SS Loadings	% Variance	*Cum*. %	Key Regions
RC1: Global/Lateral PFC	7.11	32.3%	32.3%	Sup. Frontal, Caudal/Rostral Mid-Frontal, Pars Opercularis
RC2: Orbitofrontal	2.87	13.1%	45.4%	Lateral OFC, Medial OFC, Pars Orbitalis
RC3: Anterior Cingulate	1.83	8.3%	53.7%	Rostral ACC, Caudal ACC (bilateral)

PCA, principal component analysis; SS, sum of squared loadings; Cum., cumulative; RC, rotated cortical thickness component; PFC, prefrontal cortex; OFC, orbitofrontal cortex; ACC, anterior cingulate cortex; Sup., superior; Mid-Frontal, middle frontal.

Global/Lateral PFC Thickness (RC1) had the highest SS loading (7.11; 32.3% variance) (see Figure 1A and structural covariance matrix in Figure 2). This component included superior frontal, caudal middle frontal, rostral middle frontal and pars opercularis thickness. Orbitofrontal Thickness (RC2) had the second highest SS loading (2.87; 13.1% variance) and loaded primarily onto thickness indices of lateral OFC and medial OFC, as well as pars orbitalis. Anterior Cingulate Thickness (RC3) had the third highest SS loading (1.83; 8.3% variance) and loaded onto rostral and caudal ACC bilaterally. The three-cluster organization of the PFC is also largely supported by the Ward D2 hierarchical clustering of the prefrontal covariance matrix displayed in Figure 2. The three components are centered on lateral PFC, OFC, and ACC, consistent with the anatomical prediction of H1, although parallel analysis favored two components (see Hypothesis Summary). Across 1,000 bootstrap resamples with one-to-one component matching, median Tucker’s congruence with the full-sample solution was 0.99 for RC1 (95% interval 0.70–1.00), 0.97 for RC2 (0.49–0.99), and 0.98 for RC3 (0.87–0.99); congruence fell below 0.85 in 19.4% of resamples for RC1 and 28.0% for RC2, but in only 0.7% for RC3 (Supplementary Table S2).

**Figure 2:**
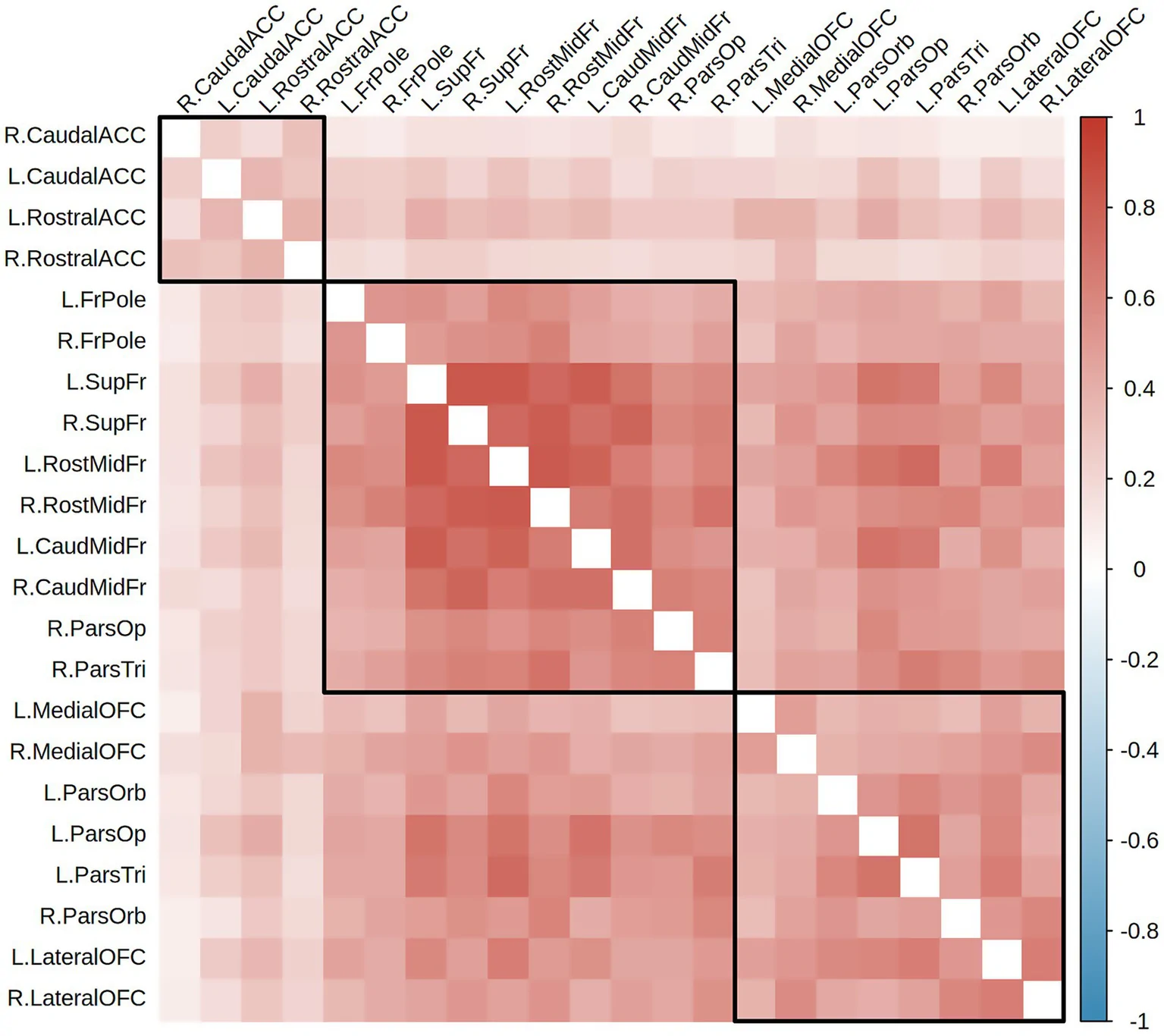
Structural covariance matrix. All 22 prefrontal thickness variables (Ward D2 hierarchical clustering) are included. Black rectangles delineate the three Ward D2-derived clusters: Cingulate (top-left, 4 regions), Global/Lateral PFC (middle, 10 regions), and Orbitofrontal (bottom-right, 8 regions). These clusters largely correspond to the PCA solution, except that left pars opercularis and left pars triangularis cluster with the orbitofrontal regions but load primarily on RC1 in the PCA (Figure 1A). Pearson correlation magnitude is reflected by color. PCA, principal component analysis; PFC, prefrontal cortex.

Inter-component correlations derived from the oblimin rotation are shown in Figure 3A. RC1 and RC2 were moderately correlated (*r* = 0.60), possibly reflecting the anatomical proximity of lateral PFC and OFC. RC1 and RC3 were correlated at *r* = 0.29 and RC2 and RC3 at *r* = 0.22. The VIFs were below 1.7 for all variables (Table 3).

**Figure 3:**
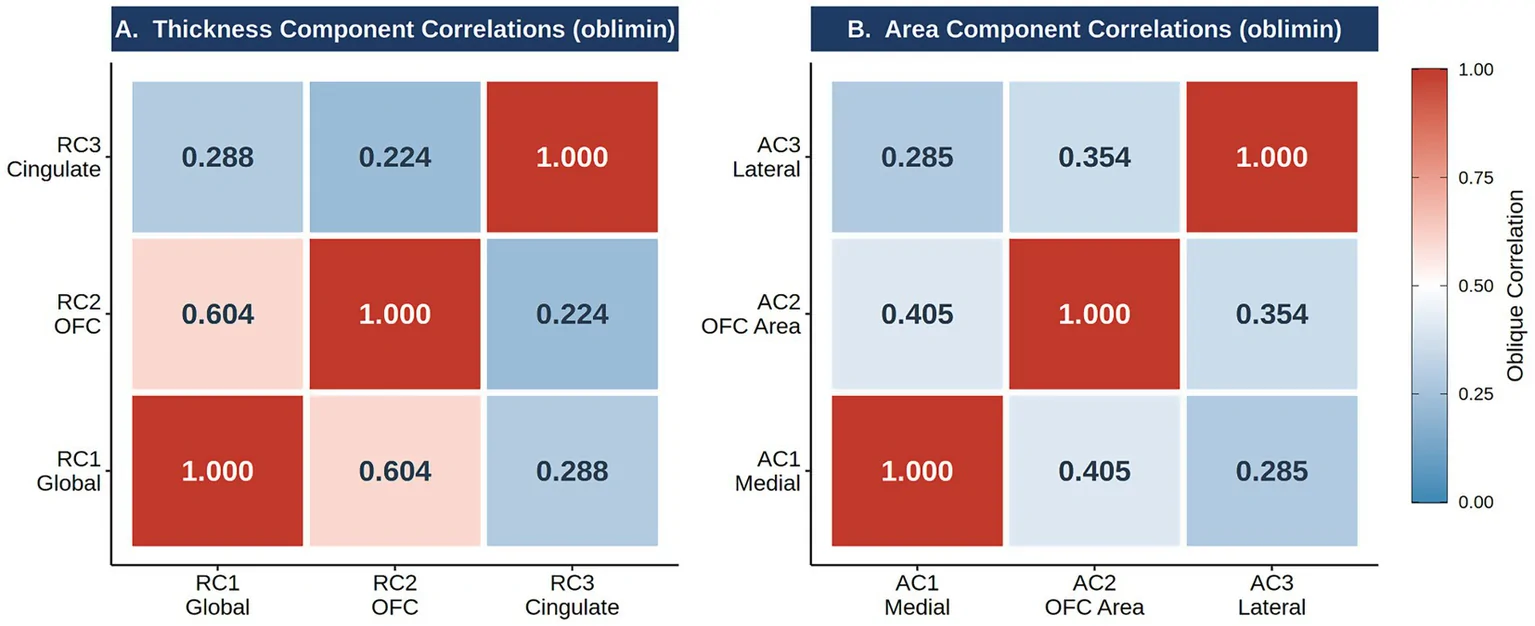
Inter-component correlations from oblique (Oblimin) PCA rotation. **(A)** Thickness components (RC1–RC3). **(B)** Surface area components (AC1–AC3). PCA, principal component analysis; RC and AC as defined in Figure 1.

#### Surface area components

PCA was performed on the 22 residualized surface area variables, which resulted in three components that explained 39.9% of the total variance and a somewhat different topographic pattern relative to the thickness solution. Component AC1 (Medial/Cingulate Surface Area) loaded the medial wall and cingulate gyrus, as well as caudal and rostral middle frontal and frontal pole surface area. Component AC2 (Orbitofrontal Surface Area) was centered on the OFC and the pars orbitalis, with additional loadings from superior frontal surface area. Component AC3 (Lateral Inferior Frontal Surface Area) was centered in inferior lateral PFC and included parts of Broca’s area (see the factor loading heatmap in Figure 1B and inter-component correlations shown in Figure 3B). The thickness and surface area solutions corresponded only partially (Tucker’s congruence up to 0.81 for RC2 and AC2 and 0.68 for RC1 and AC1; Supplementary Table S2), consistent with their largely independent genetic influences (Panizzon et al., 2009).

### Regression results: cortical thickness models

#### Inhibitory control: flanker task

Regression of Flanker Age-Adjusted Score onto RC1, RC2, RC3, age and sex was statistically significant, *F*(5, 737) = 4.13, *p* = 0.001, R^2^ = 0.027. The full model coefficients are reported in Table 5, including unstandardized and standardized coefficients, confidence intervals and partial eta-squared effect sizes for each predictor. The forest plot of standardized coefficients from the Flanker, Gambling RT Difference, and 2-Back RT models is shown in Figure 4, and the brain-behavior relationship scatter plots are shown in Figure 5.

**Table 5:** Multiple regression for Flanker age-adjusted score and thickness components (RC1, RC2, RC3) + age + sex.

Predictor	*B* [95% CI]	*t*	*p*	β	η^2^p
(Intercept)	102.23 [96.40, 108.07]	34.39	<0.001	—	—
RC1: Global/Lateral PFC	−1.02 [−1.93, −0.12]	−2.22	0.027	−0.103	0.007
RC2: Orbitofrontal	0.33 [−0.56, 1.22]	0.73	0.467	0.033	0.0007
RC3: Anterior Cingulate	−0.44 [−1.18, 0.30]	−1.18	0.240	−0.045	0.0019
Age (midpoint)	−0.03 [−0.23, 0.16]	−0.35	0.727	−0.013	0.0002
Sex (male = 1)	2.43 [0.99, 3.87]	3.32	<0.001	+0.122	0.015

B, unstandardized coefficient; CI, confidence interval; t, t statistic; β, standardized coefficient; η^2^p, partial eta-squared; RC, rotated cortical thickness component; PFC, prefrontal cortex. *p* values are uncorrected. Benjamini–Hochberg false discovery rate-adjusted value for RC1: *q* = 0.054 across the four hypothesis tests (H2–H5); *q* = 0.162 across all 12 thickness component tests; *q* = 0.216 across all 24 component tests (thickness and surface area).

**Figure 4:**
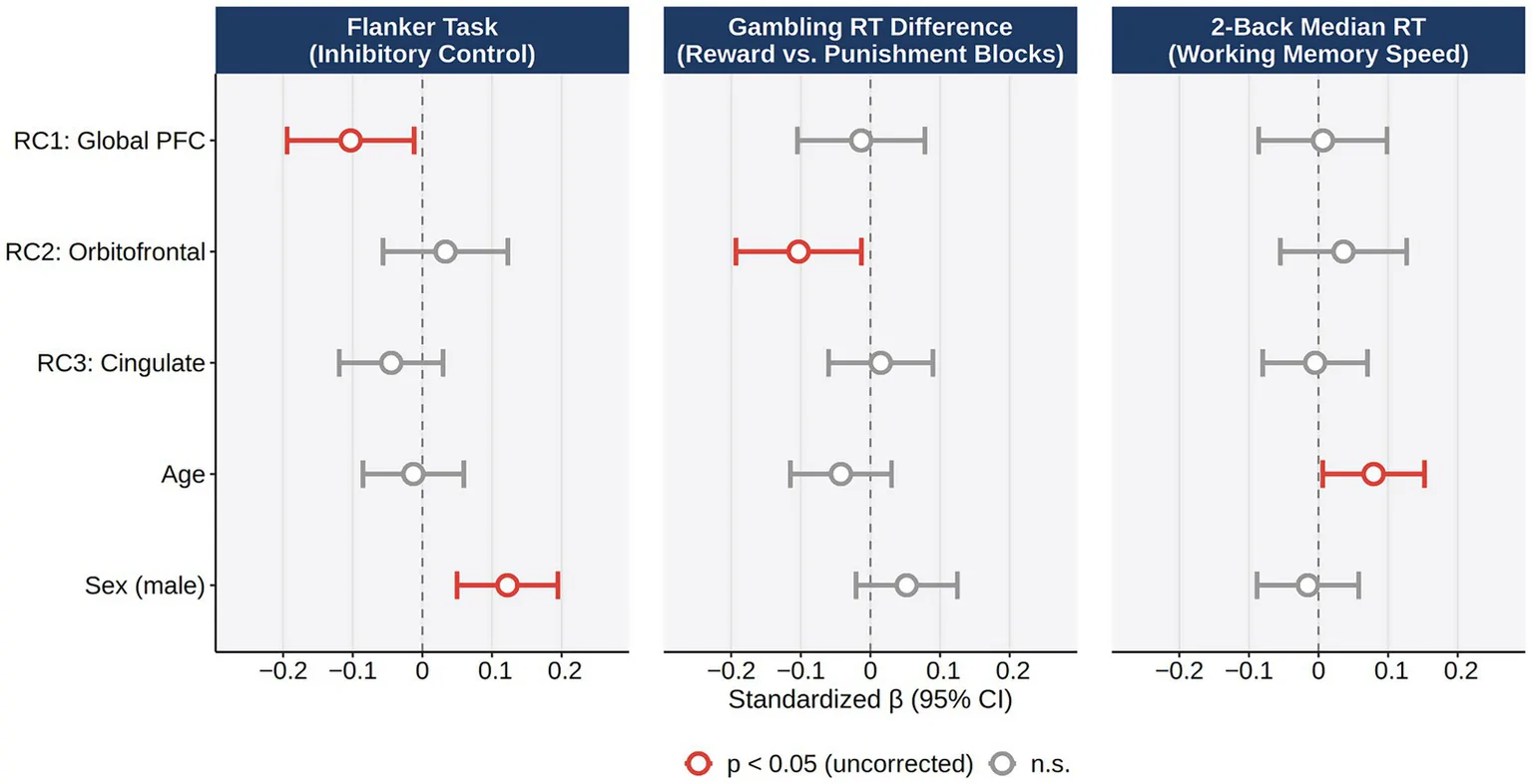
Forest plot of standardized regression coefficients. Coefficients (*β*) with 95% confidence intervals are shown for the Flanker, Gambling RT Difference, and 2-Back RT models. No structural coefficient survived Benjamini–Hochberg FDR correction. Red = *p* < 0.05 (uncorrected); gray = non-significant; dashed vertical line = zero effect. RT, reaction time; RC as defined in Figure 1.

**Figure 5:**
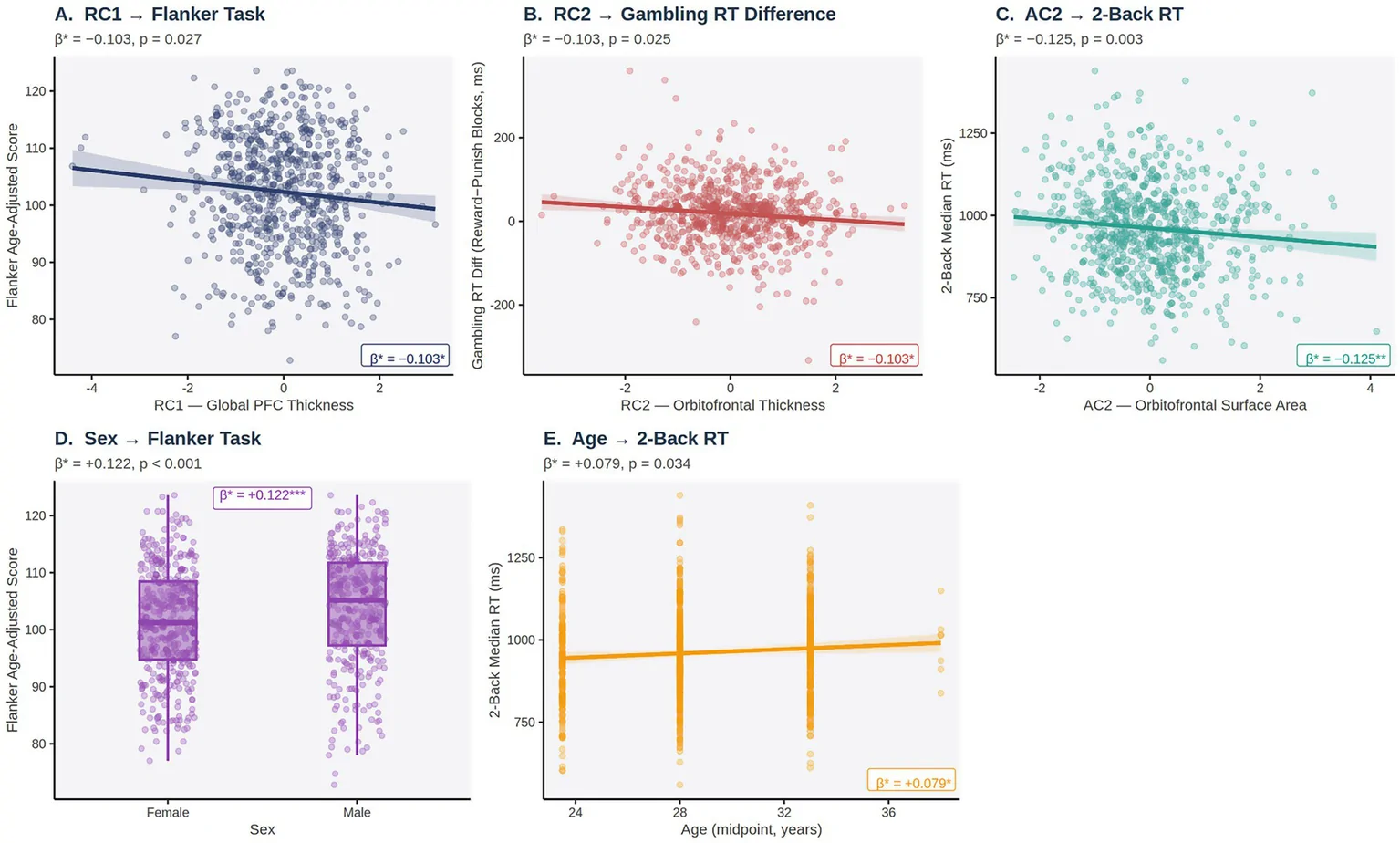
Brain–behavior scatter plots. **(A)** RC1 (global PFC thickness) related to Flanker inhibitory control. **(B)** RC2 (orbitofrontal thickness) predicting gambling RT difference. **(C)** AC2 (orbitofrontal surface area) related to 2-back RT. **(D)** Sex effects on Flanker performance. **(E)** Age related to 2-back RT. Shaded bands show 95% confidence intervals. β*, standardized coefficient; *p < 0.05; **p < 0.01; ***p < 0.001; n.s., non-significant (all uncorrected). Of the structural associations shown, only AC2 survived FDR correction, and only within the surface area family (*q* = 0.032; *q* = 0.064 across all 24 component tests). PFC, prefrontal cortex; RT, reaction time; RC and AC as defined in Figure 1.

The only structural factor that nominally predicted the inhibitory control scores in the full model was RC1 (Global/Lateral PFC Thickness): *B* = −1.02, 95% CI [−1.93, −0.12], β = −0.103, *t*(737) = −2.22, *p* = 0.027, η^2^p = 0.007 (see Table 5, Figure 4, and the RC1-Flanker scatter in Figure 5A). This association did not survive FDR correction (*q* = 0.054 across the four hypothesis tests; *q* = 0.162 across all 12 thickness component tests). The negative coefficient indicates that greater expression of the lateral PFC thickness component was associated with lower inhibitory control scores; possible interpretations are considered in the Discussion. RC1 correlated strongly with whole-cortex mean thickness (*r* = 0.85). With mean thickness added as a covariate, the RC1 coefficient decreased by about 30% (*B* = −0.72 vs. −1.02) while its standard error rose from 0.46 to 0.72 (VIFs 4.00 for RC1 and 4.90 for mean thickness), so the association was no longer significant (β = −0.073, *p* = 0.32); mean thickness was also non-significant in that model (*p* = 0.59), although on its own it predicted Flanker scores (*B* = −11.08, *p* = 0.012). These mean thickness models were added at review, were not prespecified, and are uncorrected; results were similar with mean thickness residualized on age, sex, and ICV (B = −12.48, *p* = 0.005) and with robust regression (B = −11.65, *p* = 0.011). When mean thickness was instead regressed out of the regional thickness measures before PCA, the RC1 association was not significant (β = −0.036, *p* = 0.33). The RC1 association was otherwise similar with HC3 standard errors (*p* = 0.022), robust regression (*p* = 0.028), and after exclusion of 30 influential cases (*p* = 0.025), but was weaker with the unadjusted Flanker score (β = −0.085, *p* = 0.065) and with ICV omitted from residualization (*p* = 0.051) (Supplementary Table S3).

The partial η^2^ for the structural model is displayed in the effect size plot shown in Figure 6. RC1 had the largest effect size among the structural predictors in the Flanker model (η^2^p = 0.007), although this falls below the conventional small-effect benchmark of η^2^p = 0.01 (Cohen, 1988). RC2 and RC3 were not significant (*ps* > 0.23), as confirmed by their 95% CIs crossing zero in Figure 4. Interestingly, sex proved to be a further highly significant independent predictor of the age-adjusted inhibitory control score Flanker; β = +0.122, *p* < 0.001, η^2^p = 0.015 (Figure 5D) with men scoring on average about 2.4 points more than women. The age factor did not attain significance (*p* = 0.73).

**Figure 6:**
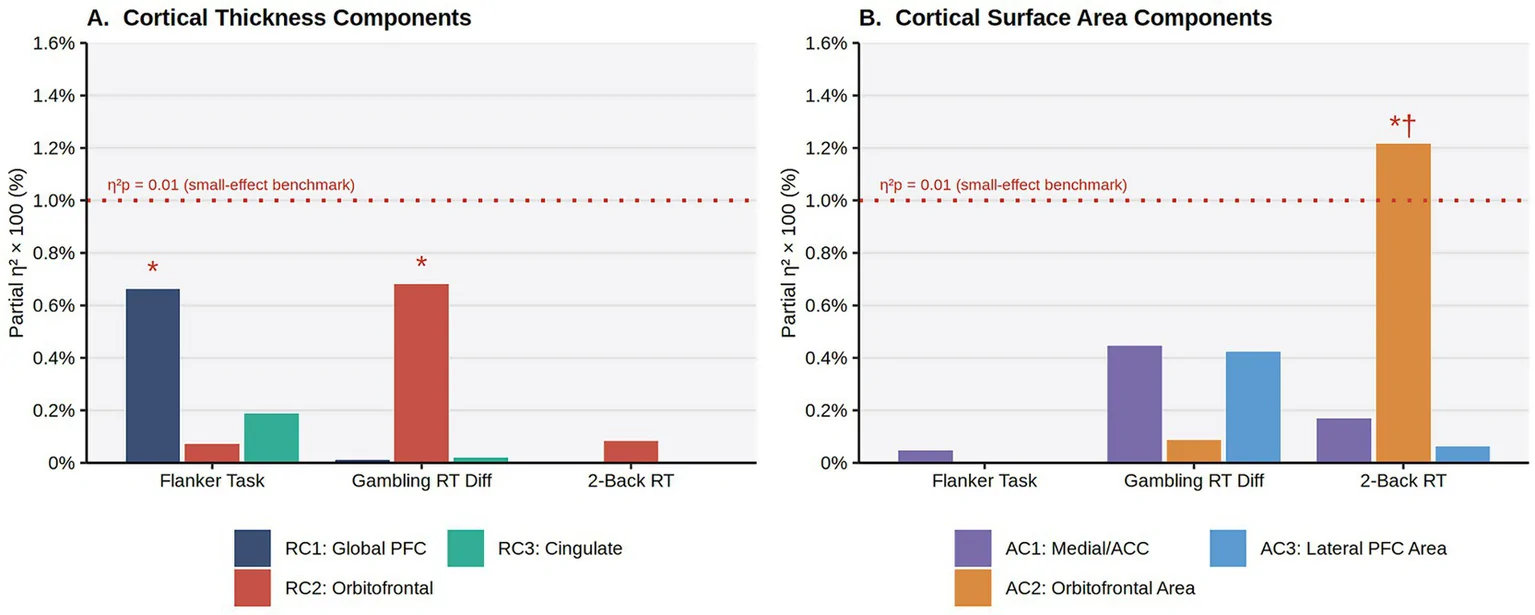
Partial *η*^2^ effect sizes by outcome and component. **(A)** Cortical thickness components (RC1–RC3). **(B)** Surface area components (AC1–AC3). Asterisk (*) denotes uncorrected *p* < 0.05; dagger (†) denotes *q* < 0.05 within the 12-test family for that morphometric measure. Dotted red line marks the conventional small-effect benchmark (*η*^2^*p* = 0.01; Cohen, 1988). No thickness effect survived FDR correction; the AC2 effect on 2-Back RT survived within the surface area family but not across all 24 component tests (*q* = 0.064). Block Choice Score results are in Supplementary Tables S5, S6. *η*^2^*p*, partial eta-squared; RC and AC as defined in Figure 1.

#### Reward-punishment block context: gambling task

Regression of Gambling RT Difference (reward-block minus punishment-block RT for “larger” guesses) on thickness components was significant, *F*(5, 737) = 2.58, *p* = 0.025, R^2^ = 0.017. Full model results are displayed in Table 6; the RC2 coefficient is depicted in the forest plot in Figure 4, and the brain-behavior scatter is shown in Figure 5B.

**Table 6:** Multiple regression for gambling RT difference (reward-block − punishment-block ms, “larger” guesses) and thickness components (RC1, RC2, RC3) + age + sex.

Predictor	*B* [95% CI]	*t*	*p*	β	η^2^p
(Intercept)	38.77 [−3.40, 80.93]	1.81	0.071	—	—
RC1: Global/Lateral PFC	−0.95 [−7.48, 5.58]	−0.29	0.775	−0.013	0.0001
RC2: Orbitofrontal	−7.35 [−13.77, −0.93]	−2.25	0.025	−0.103	0.007
RC3: Anterior Cingulate	1.05 [−4.29, 6.39]	0.39	0.699	0.015	0.0002
Age (midpoint)	−0.83 [−2.24, 0.59]	−1.14	0.253	−0.042	0.0018
Sex (male = 1)	7.43 [−2.96, 17.83]	1.40	0.161	+0.052	0.0027

RT, reaction time; B, unstandardized coefficient; CI, confidence interval; t, t statistic; β, standardized coefficient; η^2^p, partial eta-squared; RC, rotated cortical thickness component; PFC, prefrontal cortex. *p* values are uncorrected. Benjamini–Hochberg false discovery rate-adjusted value for RC2: *q* = 0.054 across the four hypothesis tests (H2–H5); *q* = 0.162 across all 12 thickness component tests; *q* = 0.216 across all 24 component tests (thickness and surface area).

RC2 (OFC thickness) was the only nominally significant predictor: *B* = −7.35, 95% CI [−13.77, −0.93], β = −0.103, *t*(737) = −2.25, *p* = 0.025, η^2^p = 0.007 (see Table 6; Figure 6). This association did not survive FDR correction (*q* = 0.054 across the four hypothesis tests; *q* = 0.162 across all 12 thickness component tests). Individuals with greater expression of the OFC thickness component showed a smaller RT difference between reward and punishment blocks. The corresponding association for “smaller” guesses was in the same direction but not significant (β = −0.053, *p* = 0.25; Supplementary Table S1). The RC2 association remained nominally significant with mean cortical thickness as a covariate (β = −0.098, *p* = 0.046; r between RC2 and mean thickness = 0.66), with HC3 standard errors (*p* = 0.027), and with ICV omitted from residualization (*p* = 0.027), but not with robust regression (*p* = 0.13) or after exclusion of 36 influential cases (β = −0.066, *p* = 0.17). It was also not significant when mean thickness was regressed out of the regional measures before PCA (β = −0.052, *p* = 0.16) (Supplementary Table S3). Mean thickness alone also predicted the Gambling RT Difference (B = −69.35, *p* = 0.029; with mean thickness residualized on age, sex, and ICV, *p* = 0.030), but not under robust regression (B = −30.09, *p* = 0.31), the estimator under which the RC2 association was also lost. These mean thickness models were added at review, were not prespecified, and are uncorrected. The modest negative slope with a high degree of scatter surrounding the trend line is depicted in Figure 5B. The other structural covariance measures (RC1, RC3) were not significant (*ps* > 0.69; Figure 4 confirms their CI crosses zero). Sex and age were also not significant.

#### Block choice score

Because the BCS could not test H4 (see Cognitive Outcome Variables), its models are reported in Supplementary Tables S5, S6. No thickness or surface area component predicted the BCS (all *ps* > 0.09), and the RC1 × RC3 interaction was non-significant (*p* = 0.615). The mean BCS was 0.998 (IQR = 0.92–1.08).

#### Working memory speed: 2-Back task

Neither prefrontal thickness nor thickness component predicted 2-Back median RT (RC1: *p* = 0.893; all *ps* > 0.43) and the model was non-significant, *F*(5, 737) = 1.26, *p* = 0.281, R^2^ = 0.008; all less than the small effect size threshold of η^2^p = 0.01, shown in Figure 6. In the working memory model, only age predicted 2-Back median RT (β = +0.079, *p* = 0.034; Figure 5E), indicating, consistent with Salthouse (1996), that older individuals had slower working memory reaction times. This null result is inconclusive. In secondary models added at review, RC1 was associated with lower 2-Back accuracy (β = −0.135, *p* = 0.003; *q* = 0.010 across the six accuracy component tests). This association remained with mean thickness controlled (β = −0.308, *p* < 0.001), in which model mean thickness took the opposite sign (β = +0.248, *p* = 0.002; VIFs 4.00 and 4.90), a pattern consistent with suppression between highly correlated predictors; mean thickness alone did not predict accuracy (*p* = 0.37) (Supplementary Table S7). 2-Back accuracy was not a prespecified outcome, and, like the AC2 accuracy result below, this association is not used as support for any conclusion.

### Regression results: surface area models

Surface area components did not predict Flanker scores, the Gambling RT Difference, or the BCS (all *ps* > 0.06; Supplementary Table S5). For 2-Back RT, the surface area model was significant, *F*(5, 737) = 2.86, *p* = 0.014, R^2^ = 0.019: greater orbitofrontal surface area (AC2) was associated with faster 2-Back RT (*B* = −17.80, 95% CI [−29.40, −6.20], β = −0.125, *t*(737) = −3.01, *p* = 0.003, η^2^p = 0.012; Figure 5C). This association survived FDR correction within the 12 surface area component tests (*q* = 0.032) but not across all 24 component tests (*q* = 0.064), and it was not prespecified. AC2 correlated r = 0.38 with total cortical surface area, and the association remained with total surface area as a covariate (β = −0.102, *p* = 0.019), with HC3 standard errors (*p* = 0.005), with robust regression (*p* = 0.004), after exclusion of 46 influential cases (β = −0.126, *p* = 0.003), and with ICV omitted from residualization (β = −0.151, *p* < 0.001) (Supplementary Table S4). Greater AC2 expression was also associated with higher 2-Back accuracy (β = +0.150, *p* < 0.001; *q* = 0.001 across the six accuracy component tests), and the association with 2-Back RT remained, attenuated, with accuracy as a covariate (β = −0.090, *p* = 0.028); these results are used only to rule out a speed-accuracy tradeoff as the explanation for the RT association (Supplementary Table S7). However, when total surface area was regressed out of the regional surface area measures before the PCA was repeated, the reconstructed AC2 closely matched the original (Tucker’s congruence 0.91), but its association with 2-Back RT was attenuated by about half and was no longer significant (β = −0.065, *p* = 0.078) (Supplementary Table S4). Total surface area alone, entered without the components alongside age and sex, was associated with faster 2-Back RT (β = −0.100, *p* = 0.032), and its coefficient in the model that also included AC2 was β = −0.106 (*p* = 0.070); with total surface area first residualized on age, sex, and ICV, it was not associated with 2-Back RT (*p* = 0.37) (Supplementary Table S4). The AC2 association therefore could not be cleanly separated from global surface area, but global surface area did not account for it.

### Hypothesis summary

In brief, H1 was only partially supported: the three components were anatomically interpretable, but parallel analysis favored two components, the three-component solution was retained by decision rather than indicated by parallel analysis, and RC1 and RC2 reorganized in a substantial minority of bootstrap resamples. H2 was not supported: greater expression of the lateral PFC component was nominally associated with lower, not higher, Flanker scores, an unpredicted finding that did not survive FDR correction. H3 (lateral PFC predicts 2-Back RT) was not supported. H4 could not be tested, because the BCS is block-level and does not capture trial-to-trial responses to feedback. H5 could not be tested as stated, because the Gambling RT Difference compares the same guess across block contexts; RC2 was nominally associated with a smaller block-context RT difference, which did not survive FDR correction. The surface area association with 2-Back RT was not covered by any hypothesis.

## Discussion

### Overview of findings

Three main points can be derived from this study. First, the present analysis found that prefrontal thickness separates into covariance components whose anatomical layout resembles subdivisions described in prior work; correspondence with functional networks was not tested. Second, two thickness components showed nominal associations with two of the outcomes (lateral PFC thickness with Flanker scores and OFC thickness with the Gambling RT Difference), but neither survived correction for multiple comparisons or the full set of sensitivity analyses. Third, an unpredicted association between greater orbitofrontal surface area and faster 2-Back RT survived correction within its family and most sensitivity analyses, but was attenuated when total surface area was regressed out before PCA.

Taken together, these data are consistent with a more complex relationship between brain structure and cognition than can be accommodated by a general theory of prefrontal function, but they do not establish one. The two nominal associations each explained less than 1% of behavioral variance and did not survive FDR correction, and brain–behavior associations of this size are unstable in samples of this scale and tend to be inflated when selected on statistical significance (Marek et al., 2022). They are therefore best treated as candidate effects for replication rather than as evidence that individual cognitive abilities, rather than some general capacity for executive function, are supported by distinct prefrontal networks. The AC2 association was somewhat larger (η^2^p = 0.012) but is subject to the same caution.

### Structural organization of the prefrontal cortex

Principal component analysis (PCA) of cortical thickness identified a three-component solution consisting of a large lateral PFC component (RC1), an orbitofrontal component (RC2), and an anterior cingulate component (RC3) that explained approximately 53.7% of the variance. Although the number of components was set by decision rather than by parallel analysis, the location of the cortical components is topologically consistent with known anatomical and functional subdivisions of the PFC.

Classic cytoarchitectonic views of brain structure have long posited the existence of medial, orbital, and lateral systems of prefrontal cortex. For example, Petrides (2005) noted distinct lateral prefrontal circuits were involved in different aspects of executive control, while Öngür and Price (2000) noted orbitofrontal and medial networks involved in emotion and value. That a data-driven decomposition yields similar divisions is consistent with prior anatomy but is not evidence of distinct biological networks, because spatially adjacent regions covary in morphometry partly through shared reconstruction error and spatial autocorrelation.

A notable finding is the moderate correlation between the lateral and orbitofrontal components (*r* = 0.60), which could potentially obscure the distinctiveness of each component. This overlap is anatomically expected when one considers the anatomy of the frontolimbic circuits (Haber and Behrens, 2014). The lateral PFC and OFC are heavily interlinked through circuits such as the uncinate fasciculus, and they both share connections with limbic regions that mediate valuation and emotion processing. Structural covariance between neighboring components is therefore expected.

Although the components were moderately correlated, this did not translate into statistical redundancy. In the primary models, the regression diagnostics showed no evidence of high multicollinearity (all VIFs ≤ 1.64, although VIFs reached 4.90 in the mean thickness sensitivity models), so each component’s association with cognition could be estimated separately when included in the models. An oblimin rotation was used, which allows for correlation between components, to preserve the anatomical relationships between brain regions, which are arguably real and meaningful rather than artificially orthogonal.

### Surface area versus cortical thickness

Cortical surface area and thickness showed only partly overlapping covariance structure in this analysis. The cortical surface area PCA explained only 39.9% of the variance in the data and showed only partial correspondence with the thickness solution (largest Tucker’s congruence 0.81, between RC2 and AC2). Furthermore, apart from the unpredicted association between orbitofrontal surface area and 2-Back RT, no surface area component predicted the outcomes examined.

A potential concern is that the separation of cortical thickness and surface area into distinct components may not always be biologically valid. Previous work has shown that cortical thickness and surface area reflect different aspects of the brain (Panizzon et al., 2009). Indeed, Panizzon et al. (2009) used twin data to show that cortical thickness and surface area are determined by largely independent sets of genes and that they follow distinct developmental trajectories. They found that surface area is typically determined during early stages of cortical development, particularly during the early growth phase of the brain in the prenatal and early postnatal periods. In contrast, cortical thickness is more susceptible to changes during later developmental processes such as synaptodendritic plasticity and myelination.

The orbitofrontal surface area association with 2-Back RT survived correction within the surface area family and most sensitivity analyses, and it was not explained by a speed-accuracy tradeoff. It was nevertheless unpredicted, did not survive correction across all 24 component tests, and was small (η^2^p = 0.012). It was also attenuated by about half and no longer significant when total surface area was regressed out of the regional measures before PCA, and it strengthened when ICV was omitted from residualization (β = −0.151), indicating that part of the signal shares variance with overall cortical and head size. Total surface area alone was associated with 2-Back RT only before adjustment for ICV, so these results show that the AC2 association cannot be cleanly separated from global surface area, not that global surface area explains it. Because surface area largely reflects early expansion of the cortical sheet (Raznahan et al., 2011), one possibility is that it indexes a developmental difference relevant to working memory, but its dependence on global size means that a regionally specific orbitofrontal account is not supported by the present data. Because the thickness and surface area associations were not compared directly, the data do not establish that thickness and surface area differ in behavioral relevance.

### Lateral prefrontal thickness and inhibitory control

One of the more surprising results was the relationship between lateral PFC thickness and inhibitory control. The regression analysis found a nominal negative association between the lateral thickness component (RC1) and performance on the Flanker task. People with thicker lateral PFC networks had slightly poorer performance on the task. However, RC1 correlated strongly with whole-cortex mean thickness (*r* = 0.85). Adding mean thickness reduced the RC1 coefficient by about 30% but increased its standard error by more than half, and mean thickness alone also predicted Flanker scores, so the present data cannot distinguish a global thickness effect from one specific to lateral PFC. Regressing mean thickness out of the regional measures before PCA did not resolve this, because the RC1 association was then non-significant. For Flanker scores, mean thickness alone predicted performance, including under robust regression, and the RC1 association did not survive regressing mean thickness out before PCA, so the more parsimonious account is that greater global cortical thickness is associated with somewhat poorer Flanker performance in this sample. For the Gambling RT Difference, the mean thickness association was lost under robust regression (*p* = 0.31), as the RC2 association was, so neither a global nor an orbitofrontal account is supported. In neither case is a prefrontal-specific thickness effect demonstrable. These mean thickness models were added at review, were not prespecified, and their *p* values are uncorrected.

Why would more cortex be associated with poorer performance? Generally, it was assumed that greater resources result in greater ability. Several explanations are possible, and because this association did not survive correction for multiple comparisons, they are offered provisionally. A developmental perspective holds that the brain increases in efficiency by removing unnecessary connections (synaptic pruning) and reducing cortical thickness while still supporting optimal functioning (Shaw et al., 2006). However, this account is less directly applicable in a sample aged 22–36, in whom developmental pruning is largely complete.

An alternative explanation concerns adult microstructure and measurement. Greater intracortical myelination can shift the FreeSurfer gray/white boundary outward and reduce estimated thickness (Natu et al., 2019), a pattern shown in visual cortex during childhood; in adults, frontal intracortical myelin continues to increase into the late 30s (Grydeland et al., 2013). A relatively thin lateral PFC may therefore partly reflect greater myelin content rather than less cortical tissue. Partial-volume effects during surface reconstruction and individual differences in surface geometry may also contribute. Other studies have shown similar relationships between lower cortical thickness and better performance on executive function tasks in young adults (Tamnes et al., 2010), but the present data cannot distinguish among these possibilities.

Males also had higher age-adjusted Flanker scores. Because NIH Toolbox Flanker scores are not sex-adjusted and incorporate reaction time once accuracy criteria are met (Zelazo et al., 2013), a general male advantage in response speed is a plausible explanation, rather than a difference in inhibitory control per se. The effect of sex is also controlled for in the process of structural residualization, and so does not contaminate the association between RC1 and inhibition.

### Orbitofrontal thickness and reward-punishment context effects on response time

A negative association between OFC thickness (RC2) and the Gambling RT Difference means that individuals with greater RC2 expression showed a smaller difference in response speed between reward and punishment blocks for the same guess. Because block type is not cued before each guess, this reflects how much recent outcome context carries over into response speed, not how participants respond to larger rewards versus larger punishments.

This association was lost under robust regression (*p* = 0.13) or after exclusion of 36 influential cases (when the coefficient roughly halved, *B* = −3.90), and it was not significant for “smaller” guesses (*p* = 0.25) or when mean thickness was regressed out before PCA (*p* = 0.16). One possibility, consistent with accounts of the OFC as maintaining outcome expectancies that are updated by feedback (Schoenbaum et al., 2009; Wallis, 2007), is that greater RC2 expression relates to response speed that is less sensitive to recent outcome history; given these results, the association should not be interpreted further.

Greater lateral PFC thickness was nominally associated with lower inhibitory control scores on the Flanker task, but it was not associated with performance on the gambling task. Conversely, greater OFC thickness was nominally associated with a smaller block-context RT difference, but it was not associated with performance on the inhibitory control task. This pattern does not establish a dissociation between the two networks, which would require a test of the difference between coefficients; that test was not performed, and neither association survived correction.

### Interpreting the null findings

The Block Choice Score could not test H4: with uncued blocks and predetermined outcomes, even a participant who followed strict win-stay and lose-switch rules would be expected to score near 1, so its null results (Supplementary Tables S5, S6) are uninformative. Trial-level choice and outcome sequences, if they can be recovered from the task fMRI packages, would be needed to test H4.

The null result for thickness and working-memory reaction time is also inconclusive. Processing speed is a network function mediated by distributed networks of brain tissue, including frontoparietal networks and white matter tracts (Kane and Engle, 2002). Processing speed declines with age (Salthouse, 1996), and white matter properties such as tract integrity may be more relevant to it than local cortical thickness. Diffusion metrics along the superior longitudinal and uncinate fasciculi, and T1w/T2w intracortical myelin maps (Glasser and Van Essen, 2011), would provide more direct structural correlates of processing speed and response inhibition than macrostructural cortical thickness alone.

### Limitations

Several limitations should be noted. First, due to the cross-sectional design, it is unclear whether there is a causal effect of structural covariance on cognition. It is possible that both structural covariance and cognition are influenced by common genetic or environmental factors. Elucidating the temporal relationships between them will require longitudinal studies to examine whether alterations in the brain are followed by alterations in cognition or vice versa. Second, the HCP sample includes twins and non-twin siblings, and family structure was not modeled because family identifiers are available only in the HCP Restricted Data release. Treating related participants as independent likely underestimates standard errors, so the reported p and *q* values are likely anti-conservative, and replication with mixed-effects models or exchangeability-block permutation (Winkler et al., 2015) is needed. Third, neither prespecified thickness association survived FDR correction, each explained less than 1% of behavioral variance (η^2^p = 0.007 for each), and the one association that survived correction within its family (AC2, η^2^p = 0.012) was not prespecified and did not survive regressing total surface area out before PCA. Brain-wide association studies indicate that effects of this magnitude are unstable in samples of fewer than several thousand participants and tend to be inflated when selected on significance (Marek et al., 2022), so the present results should be regarded as preliminary. Fourth, the HCP sample, as with all neuroimaging studies, may not be representative of all individuals. The requirement for uniformity (i.e., controlling for as many as possible confounding factors) has led to a focus on a relatively homogenous population of healthy young adults. This choice does not hold for all studies; for instance, many alterations in prefrontal brain structure may have distinct effects in clinical populations or older adults, for whom more pronounced structural changes may exhibit more pronounced effects on behavior. Fifth, education was not included as a covariate because it is available only in the restricted release. Educational attainment influences cognitive performance, and the Flanker score used here is age-adjusted but not education-adjusted; because education may also partly mediate the relationship between cortical development and adult cognition, models with and without it should be compared in future work. Sixth, several behavioral measures have psychometric limitations: the BCS is a block-level measure that does not capture trial-to-trial responses to feedback, the Gambling RT difference score is expected to have reduced reliability (Hedge et al., 2018), and 2-Back accuracy was examined only in secondary analyses. Parameters from drift-diffusion or reinforcement-learning models (Ratcliff and McKoon, 2008) would provide more reliable estimates of valuation and decision latency. Seventh, cortical thickness as calculated by FreeSurfer is only an imperfect proxy for the microstructure of the brain. Because thickness is a multi-factorial quantity that is altered by a number of biological processes (e.g., myelination, branching of neurites, and different numbers of neurons), quantifying the effects of any one process on thickness requires more than morphometry. Additional imaging approaches will be required to identify the contributors to changes in cortical thickness. The Desikan-Killiany atlas also combines cytoarchitecturally distinct prefrontal areas into coarse parcels, which limits how finely the components can be interpreted anatomically, and component scores index each participant’s expression of population-level covariance patterns rather than individual-specific structural connectivity. The three-component thickness solution was retained by decision rather than by parallel analysis, and two of its components were only moderately stable across bootstrap resamples. Finally, all values of *R*^2^ were low (≤0.027), as is typical for many morphometric studies in normal individuals. The heterogeneity of cognitive processes is large, and the structural, functional, genetic, and environmental contributors to cognitive measures have not yet been fully characterized.

### Implications and future directions

Although the present study is limited in that it does not control for disease, genotype, or a full range of cognitive measures, it illustrates how structural covariance analysis of MRI data can be applied to prefrontal morphometry, using a methodology that is as general as possible; whether this approach is more informative than analysis at the level of individual brain regions was not tested here. PCA produced thickness components with an anatomically interpretable layout, two of which showed nominal associations with cognition, and an orbitofrontal surface area component showed an association with 2-Back RT that depended in part on global surface area.

There are many potential directions for future research. The most immediate next step is to replicate the present associations, particularly the orbitofrontal surface area association with 2-Back RT, with global surface area modeled, with family structure modeled and education included, using the HCP Restricted Data release or an independent sample. Single-subject morphometric similarity networks (Seidlitz et al., 2018) would allow individual-level rather than population-level inference, and repeating the decomposition with the multimodal HCP-MMP1.0 parcellation (Glasser et al., 2016) would test whether the components hold at finer anatomical resolution, and T1w/T2w myelin maps could test the myelin account of the lateral PFC association directly. Another important step is to consider how structural covariance networks change over the course of development and across the lifespan in future longitudinal investigations. Combining cortical thickness measurements with diffusion imaging and functional connectivity data would provide a much more complete mapping of the organization of the brain. In addition, including additional tasks in the cognitive battery, such as looking at performance on capacity-based working memory tasks, working memory accuracy, and metacognitive confidence judgments in these tasks, could help differentiate between which cognitive tasks demonstrate strong structural–functional correspondence and which do not.

## Conclusion

Can information about cognition be derived from the structure of PFC? This exploratory analysis found that prefrontal thickness separates into three anatomically interpretable components, although the data favored two and the third was retained by decision. Two thickness components showed nominal associations with Flanker scores and the Gambling RT Difference, but neither survived FDR correction; whole-cortex mean thickness accounted for the Flanker association more parsimoniously than a prefrontal-specific component, and the Gambling RT Difference association was not robust under either account. An unpredicted association between greater orbitofrontal surface area and faster 2-Back RT survived correction within the surface area family and most sensitivity analyses, but could not be cleanly separated from global surface area. No prefrontal-specific association was therefore robust, and these findings are best treated as candidate effects for replication in larger, family-structured samples.

## Data Availability

Publicly available datasets were analyzed in this study. This data can be found at: https://balsa.wustl.edu/project?project=HCP_YA (HCP-Young Adult 2025 release; open-access tabular data exported 5 October 2026; free registration and acceptance of the HCP Data Use Terms required). Release documentation: https://www.humanconnectome.org/study/hcp-young-adult/document/hcp-young-adult-2025-release. Analysis code (version 1.3.0): https://doi.org/10.5281/zenodo.23275723; all versions: https://doi.org/10.5281/zenodo.23112272.
