# Supplementary material for: Prefrontal cortical morphometric covariance and cognitive task performance in healthy young adults: an exploratory analysis of Human Connectome Project data
Source: Front Hum Neurosci. 2026 Oct 22;20:1976371. doi: 10.3389/fnhum.2026.1976371 (PMC13762741; doi:10.3389/fnhum.2026.1976371)
Supplement: Supplementary file 1 [file Table_1.DOCX]

**Supplementary Material**

*Prefrontal Cortical Morphometric Covariance and Cognitive Task Performance in Healthy Young Adults: An Exploratory Analysis of Human Connectome Project Data*

Supplementary Table S1. Multiple Regression for Gambling RT Difference on "Smaller" Guesses (Reward-Block − Punishment-Block ms) and Thickness Components (RC1, RC2, RC3) + Age + Sex

| **Predictor** | **B [95% CI]** | **t** | **p** | **β** | **η²p** |
| --- | --- | --- | --- | --- | --- |
| (Intercept) | 12.57 [−29.91, 55.06] | 0.58 | 0.561 | — | — |
| RC1: Global/Lateral PFC | 4.36 [−2.20, 10.93] | 1.30 | 0.193 | 0.062 | 0.0023 |
| RC2: Orbitofrontal | −3.77 [−10.26, 2.72] | −1.14 | 0.254 | −0.053 | 0.0018 |
| RC3: Anterior Cingulate | −2.30 [−7.68, 3.08] | −0.84 | 0.401 | −0.033 | 0.0010 |
| Age (midpoint) | 0.04 [−1.39, 1.47] | 0.05 | 0.957 | 0.002 | 0.0000 |
| Sex (male = 1) | 6.25 [−4.24, 16.74] | 1.17 | 0.243 | +0.044 | 0.0019 |

N = 736. F(5, 730) = 0.76, p = 0.578, R² = 0.005. p values are uncorrected.

Supplementary Table S2. Number of Components and Stability of the Cortical Thickness PCA Solution

*(A) Parallel analysis (psych::fa.parallel, principal components, 1,000 simulated data sets)*

| **Component** | **Observed eigenvalue** | **Mean random eigenvalue** | **Exceeds random** |
| --- | --- | --- | --- |
| 1 | 10.520 | 1.320 | Yes |
| 2 | 1.582 | 1.267 | Yes |
| 3 | 1.214 | 1.226 | No |
| 4 | 1.077 | 1.191 | No |
| 5 | 0.875 | 1.160 | No |

*(B) Bootstrap stability: Tucker's congruence with the full-sample solution (1,000 resamples; one-to-one matching)*

| **Component** | **Median** | **2.5th percentile** | **97.5th percentile** | **Resamples with congruence < 0.85** |
| --- | --- | --- | --- | --- |
| RC1 | 0.99 | 0.70 | 1.00 | 19.4% |
| RC2 | 0.97 | 0.49 | 0.99 | 28.0% |
| RC3 | 0.98 | 0.87 | 0.99 | 0.7% |

In each resample, bootstrap components were matched one-to-one to the full-sample components by the permutation that maximized total absolute congruence. Congruence ≥ 0.95 indicates equivalent components and 0.85–0.94 fair similarity (Lorenzo-Seva and ten Berge, 2006).

*(C) Tucker's congruence between thickness (rows) and surface area (columns) components (absolute values)*

|  | **AC1** | **AC2** | **AC3** |
| --- | --- | --- | --- |
| RC1 | 0.68 | 0.35 | 0.61 |
| RC2 | 0.23 | 0.81 | 0.24 |
| RC3 | 0.57 | 0.04 | 0.10 |

Supplementary Table S3. Sensitivity Analyses for the Two Nominal Thickness Associations

| **Analysis** | **B** | **t** | **p** | **β** | **Notes** |
| --- | --- | --- | --- | --- | --- |
| **RC1 → Flanker Age-Adjusted Score** |  |  |  |  |  |
| Main model (OLS) | −1.02 | −2.22 | 0.027 | −0.103 | SE = 0.46 |
| + Mean cortical thickness (r = 0.85) | −0.72 | −1.00 | 0.318 | −0.073 | SE = 0.72; VIF RC1 = 4.00, mean thickness = 4.90 |
| Mean thickness term in that model | −5.21 | −0.54 | 0.587 | −0.044 |  |
| Mean thickness alone (coefficient in a model without components) | −11.08 | −2.53 | 0.012 | — | VIF = 1.03 |
| Mean thickness alone, robust M-regression (Tukey biweight) | −11.65 | −2.56 | 0.011 | — |  |
| Mean thickness regressed out before PCA | −0.36 | −0.97 | 0.331 | −0.036 |  |
| HC3 standard errors | −1.02 | −2.29 | 0.022 | — |  |
| Robust M-regression (Tukey biweight) | −1.05 | −2.19 | 0.028 | — |  |
| Excluding Cook's D > 4/N (n = 30) | −0.98 | −2.24 | 0.025 | −0.105 |  |
| Unadjusted Flanker score (age as covariate) | −0.85 | −1.85 | 0.065 | −0.085 |  |
| ICV omitted from residualization | −0.89 | −1.96 | 0.051 | −0.090 |  |
| **RC2 → Gambling RT Difference** |  |  |  |  |  |
| Main model (OLS) | −7.35 | −2.25 | 0.025 | −0.103 | SE = 3.27 |
| + Mean cortical thickness (r = 0.66) | −6.98 | −2.00 | 0.046 | −0.098 | SE = 3.50; VIF RC2 = 1.81, mean thickness = 4.90 |
| Mean thickness term in that model | −20.55 | −0.30 | 0.767 | −0.024 |  |
| Mean thickness alone (coefficient in a model without components) | −69.35 | −2.19 | 0.029 | — | VIF = 1.03 |
| Mean thickness alone, robust M-regression (Tukey biweight) | −30.09 | −1.02 | 0.306 | — | Not robust |
| Mean thickness regressed out before PCA | −3.70 | −1.39 | 0.165 | −0.052 |  |
| HC3 standard errors | −7.35 | −2.22 | 0.027 | — |  |
| Robust M-regression (Tukey biweight) | −4.62 | −1.52 | 0.128 | — |  |
| Excluding Cook's D > 4/N (n = 36) | −3.90 | −1.38 | 0.169 | −0.066 |  |
| ICV omitted from residualization | −7.19 | −2.21 | 0.027 | −0.101 |  |

N = 743 unless noted. Mean cortical thickness is the area-weighted mean across all 68 Desikan-Killiany regions (sum of regional thickness × regional surface area, divided by total surface area). In the analysis with mean thickness regressed out before PCA, the regional thickness variables were residualized on age, sex, ICV, and mean thickness, and the PCA was repeated; congruence of the resulting components with the main solution was 0.89 (RC1), 0.91 (RC2), and 0.59 (RC3). Mean thickness was entered without residualization, alongside age and sex; with mean thickness first residualized on age, sex, and ICV, the corresponding coefficients were B = −12.48 (p = 0.005) for Flanker and B = −69.33 (p = 0.030) for the Gambling RT Difference. These mean thickness models were added at review and were not prespecified. Robust-regression p values use a normal approximation. All p values are uncorrected. Mean thickness alone was also re-estimated with robust M-regression (Tukey biweight), the estimator under which the RC2 association was lost; under it, mean thickness remained associated with Flanker scores but not with the Gambling RT Difference.

Supplementary Table S4. Sensitivity Analyses for the AC2 (Orbitofrontal Surface Area) Association With 2-Back RT, and 2-Back Accuracy

| **Analysis** | **B** | **t** | **p** | **β** | **Notes** |
| --- | --- | --- | --- | --- | --- |
| **AC2 → 2-Back median RT** |  |  |  |  |  |
| Main model (OLS) | −17.80 | −3.01 | 0.003 | −0.125 | η²p = 0.012; q = 0.032 (12 surface area tests), 0.064 (24 component tests) |
| + Total cortical surface area (r = 0.38) | −14.52 | −2.35 | 0.019 | −0.102 | VIF AC2 = 1.41, total area = 2.59 |
| Total surface area term in that model | −0.84 | −1.81 | 0.070 | −0.106 | B per 1,000 mm² |
| Total surface area alone (model without components) | −0.79 | −2.15 | 0.032 | −0.100 | B per 1,000 mm²; VIF = 1.61 |
| Total surface area residualized on age, sex, and ICV, alone | −0.49 | −0.90 | 0.367 | −0.033 | B per 1,000 mm²; model without components |
| HC3 standard errors | −17.80 | −2.82 | 0.005 | — |  |
| Robust M-regression (Tukey biweight) | −17.36 | −2.87 | 0.004 | — |  |
| Excluding Cook's D > 4/N (n = 46) | −16.45 | −2.95 | 0.003 | −0.126 |  |
| ICV omitted from residualization | −21.48 | −3.39 | < 0.001 | −0.151 |  |
| Total surface area regressed out before PCA | −9.26 | −1.77 | 0.078 | −0.065 | AC2 congruence with main solution = 0.91 |
| + 2-Back accuracy as covariate | −12.83 | −2.21 | 0.028 | −0.090 | accuracy: β = −0.233, p < 0.001 |
| **2-Back accuracy (% correct) as outcome** |  |  |  |  |  |
| AC2 (surface area model) | 1.53 | 3.71 | < 0.001 | 0.150 | η²p = 0.018 |
| RC1 (thickness model) | −1.39 | −2.96 | 0.003 | −0.135 | η²p = 0.012 |

N = 743 unless noted. Total cortical surface area is the sum of all 68 Desikan-Killiany regional surface areas; it was entered without residualization, alongside age and sex. In the analysis with total surface area regressed out before PCA, the regional surface area variables were residualized on age, sex, ICV, and total surface area, and the PCA was repeated; congruence of the resulting components with the main solution was 0.66 (AC1), 0.91 (AC2), and 0.68 (AC3). Full 2-Back accuracy models are in Supplementary Table S7. 2-Back accuracy (WM_Task_2bk_Acc; mean = 83.9%, SD = 10.3) correlated r = −0.24 with 2-Back median RT. 2-Back accuracy was not a prespecified outcome; these models were added at review. The AC2 association with 2-Back RT was not prespecified. Robust-regression p values use a normal approximation. p values are uncorrected unless labeled q. Total surface area coefficients are expressed per 1,000 mm². In the residualized row, total surface area was first regressed on age, sex, and ICV. The total surface area models were added at review and were not prespecified.

Supplementary Table S5. Multiple Regressions for Each Outcome on Surface Area Components (AC1, AC2, AC3) + Age + Sex

*(A) Flanker Age-Adjusted Score: F(5, 737) = 2.43, p = 0.034, R² = 0.016*

| **Predictor** | **B [95% CI]** | **t** | **p** | **β** | **η²p** |
| --- | --- | --- | --- | --- | --- |
| (Intercept) | 102.23 [96.36, 108.10] | 34.20 | < 0.001 | — | — |
| AC1: Medial/Cingulate | 0.24 [−0.55, 1.03] | 0.60 | 0.551 | 0.024 | 0.0005 |
| AC2: Orbitofrontal | −0.01 [−0.82, 0.80] | −0.02 | 0.986 | −0.001 | 0.0000 |
| AC3: Lateral Inferior Frontal | −0.05 [−0.82, 0.72] | −0.13 | 0.895 | −0.005 | 0.0000 |
| Age (midpoint) | −0.04 [−0.23, 0.16] | −0.35 | 0.728 | −0.013 | 0.0002 |
| Sex (male = 1) | 2.43 [0.98, 3.88] | 3.30 | 0.001 | +0.122 | 0.015 |

*(B) Gambling RT Difference (ms): F(5, 737) = 2.11, p = 0.062, R² = 0.014*

| **Predictor** | **B [95% CI]** | **t** | **p** | **β** | **η²p** |
| --- | --- | --- | --- | --- | --- |
| (Intercept) | 38.77 [−3.46, 80.99] | 1.80 | 0.072 | — | — |
| AC1: Medial/Cingulate | 5.25 [−0.42, 10.92] | 1.82 | 0.070 | 0.074 | 0.0045 |
| AC2: Orbitofrontal | 2.38 [−3.43, 8.19] | 0.80 | 0.422 | 0.033 | 0.0009 |
| AC3: Lateral Inferior Frontal | −5.00 [−10.54, 0.55] | −1.77 | 0.077 | −0.070 | 0.0042 |
| Age (midpoint) | −0.83 [−2.25, 0.59] | −1.14 | 0.254 | −0.042 | 0.0018 |
| Sex (male = 1) | 7.43 [−2.98, 17.84] | 1.40 | 0.162 | +0.052 | 0.0027 |

*(C) 2-Back median RT (ms): F(5, 737) = 2.86, p = 0.014, R² = 0.019*

| **Predictor** | **B [95% CI]** | **t** | **p** | **β** | **η²p** |
| --- | --- | --- | --- | --- | --- |
| (Intercept) | 874.67 [790.41, 958.94] | 20.38 | < 0.001 | — | — |
| AC1: Medial/Cingulate | 6.44 [−4.88, 17.76] | 1.12 | 0.264 | 0.045 | 0.0017 |
| AC2: Orbitofrontal | −17.80 [−29.40, −6.20] | −3.01 | 0.003 | −0.125 | 0.012 |
| AC3: Lateral Inferior Frontal | 3.82 [−7.25, 14.88] | 0.68 | 0.498 | 0.027 | 0.0006 |
| Age (midpoint) | 3.08 [0.25, 5.92] | 2.14 | 0.033 | 0.079 | 0.006 |
| Sex (male = 1) | −4.41 [−25.19, 16.37] | −0.42 | 0.677 | −0.015 | 0.0002 |

*(D) Block Choice Score: F(5, 737) = 0.62, p = 0.684, R² = 0.004*

| **Predictor** | **B [95% CI]** | **t** | **p** | **β** | **η²p** |
| --- | --- | --- | --- | --- | --- |
| (Intercept) | 1.00 [0.92, 1.08] | 23.46 | < 0.001 | — | — |
| AC1: Medial/Cingulate | 0.00 [−0.01, 0.01] | 0.15 | 0.884 | 0.006 | 0.0000 |
| AC2: Orbitofrontal | −0.01 [−0.02, 0.00] | −1.69 | 0.091 | −0.071 | 0.0039 |
| AC3: Lateral Inferior Frontal | 0.00 [−0.01, 0.02] | 0.74 | 0.459 | 0.029 | 0.0007 |
| Age (midpoint) | 0.00 [−0.00, 0.00] | −0.02 | 0.984 | −0.001 | 0.0000 |
| Sex (male = 1) | −0.00 [−0.02, 0.02] | −0.10 | 0.919 | −0.004 | 0.0000 |

p values are uncorrected. Benjamini–Hochberg q values across the 12 surface area component tests: AC2 → 2-Back RT, q = 0.032; all other surface area tests, q ≥ 0.27.

Supplementary Table S6. Block Choice Score (BCS) Regressed on Thickness Components (RC1, RC2, RC3) + Age + Sex

| **Predictor** | **B [95% CI]** | **t** | **p** | **β** | **η²p** |
| --- | --- | --- | --- | --- | --- |
| (Intercept) | 1.00 [0.92, 1.08] | 23.47 | < 0.001 | — | — |
| RC1: Global/Lateral PFC | 0.00 [−0.01, 0.01] | 0.14 | 0.885 | 0.007 | 0.0000 |
| RC2: Orbitofrontal | 0.01 [−0.00, 0.02] | 1.36 | 0.173 | 0.063 | 0.0025 |
| RC3: Anterior Cingulate | −0.01 [−0.02, 0.01] | −0.99 | 0.322 | −0.038 | 0.0013 |
| Age (midpoint) | 0.00 [−0.00, 0.00] | −0.02 | 0.984 | −0.001 | 0.0000 |
| Sex (male = 1) | −0.00 [−0.02, 0.02] | −0.10 | 0.919 | −0.004 | 0.0000 |

F(5, 737) = 0.71, p = 0.618, R² = 0.005. BCS mean = 0.998 (IQR = 0.92–1.08). The RC1 × RC3 interaction entered to test H4 was non-significant (B = 0.0017, p = 0.615). Because block type is not cued and outcomes are predetermined by block, the BCS cannot index trial-level win-stay/lose-switch behavior, and H4 could not be tested; the BCS models are reported here for completeness. Surface area results for the BCS are in Supplementary Table S5D.

Supplementary Table S7. 2-Back Accuracy (% Correct) Regressed on Thickness and Surface Area Components + Age + Sex

*(A) Thickness components: F(5, 737) = 9.40, p < 0.001, R² = 0.060*

| **Predictor** | **B [95% CI]** | **t** | **p** | **β** | **η²p** |
| --- | --- | --- | --- | --- | --- |
| (Intercept) | 88.36 [82.42, 94.29] | 29.23 | < 0.001 | — | — |
| RC1: Global/Lateral PFC | −1.39 [−2.31, −0.47] | −2.96 | 0.003 | −0.135 | 0.012 |
| RC2: Orbitofrontal | 0.44 [−0.47, 1.34] | 0.95 | 0.343 | 0.043 | 0.0012 |
| RC3: Anterior Cingulate | 0.15 [−0.60, 0.91] | 0.40 | 0.689 | 0.015 | 0.0002 |
| Age (midpoint) | −0.22 [−0.42, −0.02] | −2.13 | 0.033 | −0.077 | 0.006 |
| Sex (male = 1) | 3.92 [2.46, 5.39] | 5.26 | < 0.001 | +0.191 | 0.036 |

*(B) Surface area components: F(5, 737) = 11.11, p < 0.001, R² = 0.070*

| **Predictor** | **B [95% CI]** | **t** | **p** | **β** | **η²p** |
| --- | --- | --- | --- | --- | --- |
| (Intercept) | 88.36 [82.45, 94.26] | 29.38 | < 0.001 | — | — |
| AC1: Medial/Cingulate | −0.08 [−0.87, 0.71] | −0.20 | 0.842 | −0.008 | 0.0001 |
| AC2: Orbitofrontal | 1.53 [0.72, 2.35] | 3.71 | < 0.001 | 0.150 | 0.018 |
| AC3: Lateral Inferior Frontal | 0.11 [−0.67, 0.88] | 0.27 | 0.789 | 0.010 | 0.0001 |
| Age (midpoint) | −0.22 [−0.42, −0.02] | −2.14 | 0.032 | −0.077 | 0.006 |
| Sex (male = 1) | 3.92 [2.47, 5.38] | 5.29 | < 0.001 | +0.191 | 0.037 |

*(C) RC1 with whole-cortex mean thickness as a covariate*

| **Term** | **B** | **t** | **p** | **β** | **Notes** |
| --- | --- | --- | --- | --- | --- |
| RC1 | −3.15 | −4.33 | < 0.001 | −0.308 | VIF = 4.00 |
| Mean thickness | 30.57 | 3.16 | 0.002 | 0.248 | VIF = 4.90 |
| Mean thickness alone (model without components) | −3.98 | −0.89 | 0.374 | — |  |

N = 743. Benjamini–Hochberg q values across the six accuracy component tests: RC1 = 0.010, RC2 = 0.685, RC3 = 0.842, AC1 = 0.842, AC2 = 0.001, AC3 = 0.842. 2-Back accuracy was not a prespecified outcome; these models were added at review. The AC2 result is used only to rule out a speed-accuracy tradeoff for the AC2 association with 2-Back RT, and neither the AC2 nor the RC1 accuracy result is used as support for any conclusion. In panel C, the RC1 coefficient strengthened and mean thickness took the opposite sign, a pattern consistent with suppression between highly correlated predictors (r = 0.85). p values are uncorrected unless labeled q.
